# Silencing subtelomeric *VSGs* by *Trypanosoma brucei* RAP1 at the insect stage involves chromatin structure changes

**DOI:** 10.1093/nar/gkt562

**Published:** 2013-06-26

**Authors:** Unnati M. Pandya, Ranjodh Sandhu, Bibo Li

**Affiliations:** Department of Biological, Geological, and Environmental Sciences, Center for Gene Regulation in Health and Disease, Cleveland State University, Cleveland, OH 44115, USA

## Abstract

*Trypanosoma brucei* causes human African trypanosomiasis and regularly switches its major surface antigen variant surface glycoprotein (VSG) to evade mammalian host immune responses at the bloodstream form (BF) stage. Monoallelic expression of BF Expression Site (BES)-linked *VSGs* and silencing of metacyclic *VSGs* (mVSGs) in BF cells are essential for antigenic variation, whereas silencing of both BES-linked and m*VSGs* in the procyclic form (PF) cells is important for cell survival in the midgut of its insect vector. We have previously shown that silencing BES-linked *VSGs* in BF cells depends on *Tb*RAP1. We now show that *Tb*RAP1 silences both BES-linked and m*VSGs* at both BF and PF stages. The strength of *Tb*RAP1-mediated BES-linked *VSG* silencing is stronger in the PF cells than that in BF cells. In addition, Formaldehyde-Assisted Isolation of Regulatory Elements analysis and MNase digestion demonstrated that depletion of *Tb*RAP1 in PF cells led to a chromatin structure change, which is significantly stronger at the subtelomeric *VSG* loci than at chromosome internal loci. On the contrary, no significant chromatin structure changes were detected on depletion of *Tb*RAP1 in BF cells. Our observations indicate that *Tb*RAP1 helps to determine the chromatin structure at the insect stage, which likely contributes to its strong silencing effect on *VSGs*.

## INTRODUCTION

Human African trypanosomiasis is caused by infection of *Trypanosoma brucei* and is inevitably fatal without treatment. Inside the human host, bloodstream form (BF) *T. brucei* cells stay in extracellular spaces and express variant surface glycoprotein (VSG) as its major surface antigen that is exposed to the host immune system (1,2). To evade the host’s immune responses, *T. brucei* cells undergo antigenic variation and regularly switch their VSG coat (3), which is essential for a persistent infection. Although there are >1000 *VSG* genes and pseudogenes in the *T. brucei* genome (4), in BF cells, VSGs are expressed exclusively from BF *VSG* Expression Sites (BESs), which are polycistronically transcribed by RNA Pol I (5). The *VSG* gene is the last gene in the BES and is located adjacent to the telomere while the BES promoter is usually 40–60 kb upstream (6). The *T. brucei* 427 strain used in this study has ∼20 nearly identical BESs (7), 14 of which carry distinctive *VSG* genes (8). However, at any moment, only one BES promoter is fully active, resulting in a single type of VSG being expressed. *VSG* monoallelic expression ensures the effectiveness of antigenic variation and is essential for *T. brucei* virulence.

*Trypanosoma brucei* is transmitted through its insect vector, tsetse (*Glossina spp.*). After ingestion by tsetse into its midgut, *T. brucei* differentiates into the procyclic form (PF) and expresses procyclins as surface glycoproteins, of which the C-terminus is resistant to protease cleavage (9). VSG is susceptible to protease degradation, and all *VSG* genes are silent in PF cells. Therefore, *VSG* silencing at the insect stage is critical for *T. brucei* to survive in its environment, but the underlying mechanism is poorly understood. Once *T. brucei* cells migrate into the salivary glands of tsetse, they differentiate into the metacyclic form, shed procyclins, express metacyclic VSGs (mVSGs) on the cell surface and acquire infectivity again. Each cell expresses one type of mVSG, but the population expresses many different mVSGs (10). This heterogeneity will presumably facilitate the population to establish an infection in mammalian hosts (11). Similar to BF VSGs, mVSGs are also transcribed from subtelomeres by RNA Pol I, but transcription of *mVSGs* is monocistronic (12,13). *mVSG* expression is silenced within a few days after the initial infection, allowing subsequent antigenic variation to take place, but the mechanisms of *mVSG* silencing have not been extensively studied.

Accumulating data have revealed that multiple mechanisms are involved in the regulation of BES-linked *VSG* monoallelic expression at the BF stage, including limited accessibility to RNA Pol I (14), restricted transcription elongation (15,16), chromatin remodeling (17–22) and telomeric silencing (23). Telomeres, located at the ends of linear chromosomes, are essential for genome stability. In several organisms including yeast, human, mouse, *T. brucei* and *Plasmodium falciparum*, telomeres form a heterochromatic structure that suppresses the transcription of subtelomeric genes (24–29). *Trypanosoma brucei* telomere DNA consists of thousands of duplex TTAGGG repeats (30). We have previously identified *Tb*TRF as the duplex telomere DNA-binding factor (31) and *Tb*RAP1 as a *Tb*TRF-interacting partner (23). Depletion of *Tb*RAP1 led to derepression of all subtelomeric silent BES-linked *VSGs* and simultaneous expression of multiple VSG proteins on the cell surface at the BF stage (23). However, whether *Tb*RAP1 has a similar *VSG* silencing function in PF cells and the mechanism of *Tb*RAP1-mediated silencing are not clear.

In this study, we found that depletion of *Tb*RAP1 by RNA interference (RNAi) led to derepression of *mVSGs* at both the BF and PF stages. Silencing of BES-linked *VSGs* in PF cells depends on *Tb*RAP1 and is stronger than that in BF cells. Furthermore, removal of *Tb*RAP1 led to a loosened chromatin structure in PF cells, particularly at the subtelomeric *VSG* loci, but not significantly in BF cells, indicating that *Tb*RAP1-mediated silencing involves modulation of chromatin structure in PF cells.

## MATERIALS AND METHODS

### Reverse transcription and quantitative reverse transcriptase-PCR

Total RNA was extracted using RNAstat 60 (Tel-Test Inc), treated with DNase I (Qiagen) and purified using RNeasy columns (Qiagen). Reverse transcription, quantitative reverse transcriptase-PCR (qRT-PCR) and quantification were performed as in (23).

### Formaldehyde-assisted isolation of regulatory elements

Formaldehyde-assisted isolation of regulatory elements (FAIRE) analyses were performed according to (32). Cells were fixed with formaldehyde before the chromatin was sonicated in a Bioruptor (Diagenode). Free DNA was extracted by phenol chloroform. Input samples were reverse cross-linked before DNA was extracted. The amount of DNA was estimated by quantitative PCR using iTaq SYBR Green Supermix with ROX in an Opticon II (Bio-Rad), and the amount of FAIRE-extracted DNA was normalized with that of the input DNA.

### Micrococcal nuclease digestion

In all, 5 × 10^7^ cells per sample were permeabilized with digitonin and treated with 1 unit of Micrococcal nuclease (MNase) (Worthington Biochemicals) for 1, 2.5 or 5 min. DNA was isolated from treated cells, separated by agarose gel electrophoresis, blotted onto a nylon membrane and hybridized with specific probes.

### Statistical analysis

*P*-values of unpaired *t*-tests were calculated using GraphPad Prism.

## RESULTS

### *Tb*RAP1 is essential for normal cell growth in PF cells

To examine the functions of *Tb*RAP1 in PF cells, we introduced an inducible *Tb*RAP1 RNAi construct in 29-13 cells that expresses the Tet repressor and the T7 polymerase (33). Several independent clones were obtained that exhibited different growth defects on induction of *Tb*RAP1 RNAi (Supplementary Figure S1A–C). To reduce the phenotypic variations often associated with clonal cell lines, we obtained a pool of *Tb*RAP1 RNAi cells (referred to as ‘PRi-pool’) for subsequent detailed analyses, and critical phenotypes were confirmed in the PRi-C2 clone (Supplementary Figures S1B, D and F and S8B).

**Figure 1.**
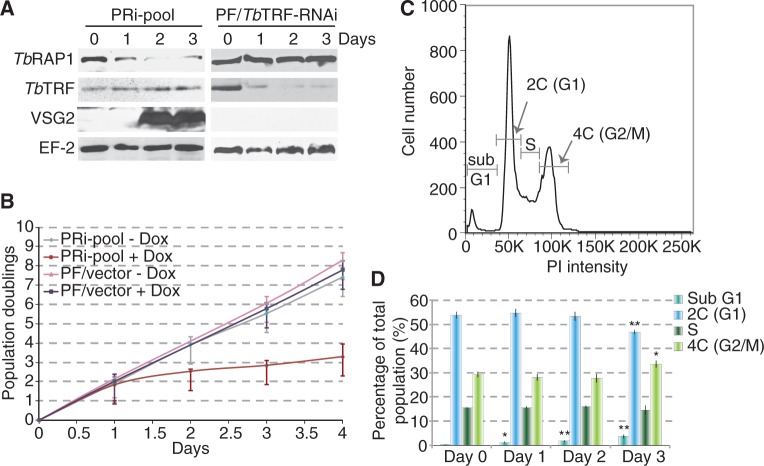
Depletion of *Tb*RAP1 led to a cell growth defect and derepression of BES-linked *VSG2* in PF cells. (**A**) Western analysis of various protein levels in PRi-pool (left) and *Tb*TRF RNAi (right) cells. Total cell lysates were prepared at days 0, 1, 2 and 3 after adding doxycycline. (**B**) Growth curves for PRi-pool and control cells with the empty vector under induced (+ Dox) or un-induced (−Dox) conditions were plotted as Population Doublings versus days after induction. Average Population Doubling values were calculated from six independent experiments. Error bars represent standard deviation. (**C**) Gating schematic of un-induced PRi-pool cells in the flow cytometry analysis. Sub-G1 cells contain DNA contents less than 2C and often experience DNA degradations. PI, propidium iodide. (**D**) Quantification of populations of PRi-pool cells at different cell cycle stages before (day 0) and after (days 1, 2 and 3) depletion of *Tb*RAP1. Unpaired *t*-tests were done to compare day 1, 2 and 3 values with the day 0 value. Significant differences are marked with asterisks. *, 0.01 < *P* ≤ 0.05; **, 0.001 < *P* ≤ 0.01.

On induction of *Tb*RAP1 RNAi, we observed a decreased *Tb*RAP1 protein level and a severe growth defect by day 2 in PRi-pool cells (Figure 1A and B, Supplementary Figure S1E), indicating that *Tb*RAP1 is essential for cell viability. Similar *Tb*RAP1 knockdown and growth arrest were seen in PRi-C2 cells (Supplementary Figure S1B and F). The control 29-13 cells carrying an empty RNAi vector did not show any growth defects or decrease in the *Tb*RAP1 protein level when treated with the same amount of doxycycline (Figure 1B, Supplementary Figure S1E). Flow cytometry analysis was performed to examine cell cycle profile after depletion of *Tb*RAP1. We observed a mild decrease in the G1 population and a mild increase in the G2/M and the sub G1 populations in PRi-pool cells (Figure 1D, Supplementary Figure S2A), which is similar to what we observed in *Tb*RAP1-depleted BF cells in our current and earlier studies (Supplementary Figure S2B and C) (23).

### BES-linked VSG silencing depends on TbRAP1 at the insect stage

**Figure 2.**
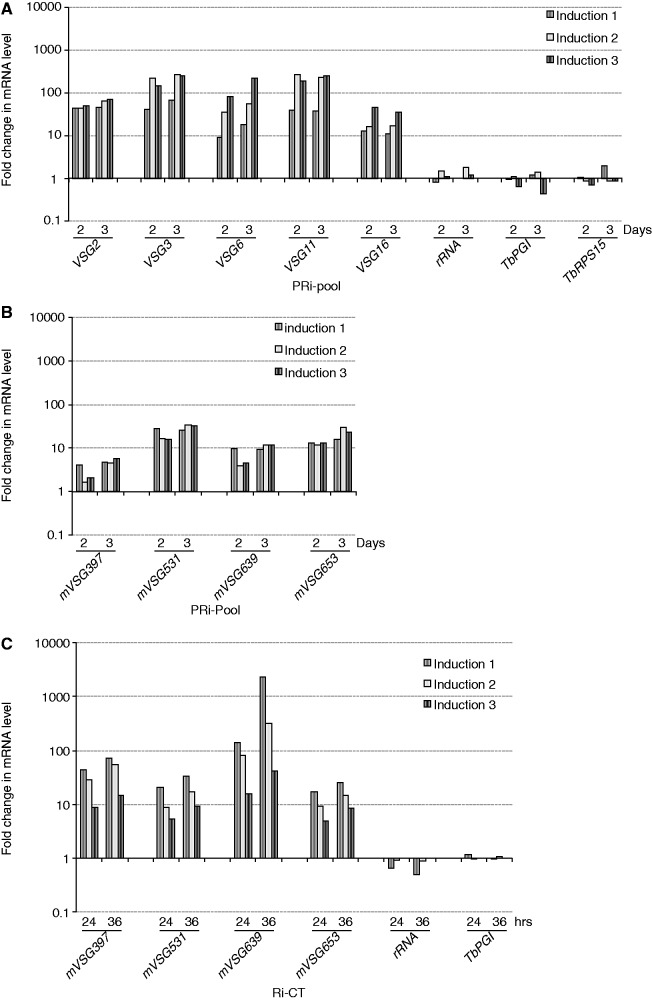
Depletion of *Tb*RAP1 resulted in derepression of BES-linked *VSGs* in PF cells (**A**) and derepression of *mVSGs* in PF (**B**) and BF (**C**) cells. Steady–state mRNA levels for BES-linked *VSGs 2*, *3*, *6*, *11*, *mVSGs 397*, *531*, *639*, *653* and control genes at days 0, 2 and 3 (PF) or at 0, 24 and 36 h (BF) after adding doxycycline in *Tb*RAP1 RNAi cells were estimated using qRT-PCR and normalized against that before induction. Day 0 value is set to 1 but not shown. The fold change in mRNA levels for three independent induction experiments was shown.

We compared the steady–state mRNA levels of several BES-linked *VSGs* before and after depletion of *Tb*RAP1 by qRT-PCR. Thirty-five percent of *Tb*RAP1 was still present at day 1 after induction of RNAi, but <10% of *Tb*RAP1 was left by day 2 (Figure 1A). We therefore examined *VSG* derepression at day 2 and day 3. All tested *VSG* mRNA levels increased several 10- to several 100-fold after depletion of *Tb*RAP1 (Figure 2A, see Supplementary Table S1 for *VSG* nomenclature). Western analysis also detected a prominent amount of VSG2 in PRi-pool cells (Figure 1A, Supplementary Figure S1E). Therefore, *Tb*RAP1 is essential for silencing BES-linked *VSGs* in PF cells. Similar to that in BF cells, expression of RNA Pol II-transcribed *Tb*PGI (a glycolytic protein), *Tb*RPS15 (a ribosomal protein) or RNA Pol I-transcribed rRNA was not affected, indicating that *Tb*RAP1’s effect on *VSGs* is specific. In addition to BES-linked and m*VSGs*, *VSG* genes located on minichromosomes (which mainly consist of internal 177 bp repeats and terminal telomere repeats) are also at subtelomeres but lack upstream promoters. Silencing of a minichromosomal *VSG*, *VSG671*, was not affected by depletion of *Tb*RAP1—we did not detect its mRNA in northern blotting (data not shown), although the *VSG671* gene was detectable in Southern analysis (Supplementary Figure S3)—indicating that *VSG* derepression requires a functional upstream promoter. Similar *VSG* derepression phenotype was also observed in PRi-C2 cells (Supplementary Figure S1D). On the contrary, no *VSG* derepression was detected in control cells by qRT-PCR (Supplementary Figure S4A) or western blotting (Supplementary Figure S1E). In addition, induction of *Tb*TRF RNAi led to a cell growth arrest within 2 days in PF cells (31), but not VSG2 expression (Figure 1A), indicating that *VSG* derepression is not a consequence of cell growth arrest. Although *Tb*RAP1 interacts with *Tb*TRF (23), it has not been proved that *Tb*TRF is strictly required for localizing *Tb*RAP1 at telomeres. Therefore, depletion of *Tb*TRF does not necessarily have the same *VSG* derepression phenotype.

### *Tb*RAP1 is required for silencing of *mVSGs* in BF and PF *T. brucei* cells

*mVSGs* are normally silent in BF and PF cells, but the silencing mechanisms are poorly understood. In our laboratory strain 427, five *mVSG* genes have been identified: *mVSGs 397*, *531*, *639*, *653* and *1954* (34). qRT-PCR analysis showed that *mVSG* mRNA levels increased several to several 10-fold on depletion of *Tb*RAP1 (Figure 2B), whereas no significant changes were detected in control cells (Supplementary Figure S4B). We performed similar analyses in BF cells using two cell lines carrying pZJMβ-based (35) *Tb*RAP1 RNAi vectors. One includes the *Tb*RAP1 BRCT fragment (Ri-BRCT) and the other the *Tb*RAP1 C-Terminus (Ri-CT). Both cell lines exhibited growth arrest and derepression of BES-linked *VSGs* on induction of *Tb*RAP1 RNAi (Supplementary Figure S5), indicating that these cells behaved similarly to the Ri-2 and Ri-9 *Tb*RAP1 RNAi cells studied previously (23). *mVSGs* were derepressed several to over a 1000-fold on *Tb*RAP1 depletion in Ri-BRCT and Ri-CT cells, but not in control cells (Figure 2C; Supplementary Figure S4C and D). Therefore, *Tb*RAP1 is required to silence *mVSGs* in both BF and PF cells.

### Comparing the *Tb*RAP1-mediated *VSG* silencing at different life cycle stages

We found that *Tb*RAP1 silences BES-linked and metacyclic *VSGs*, but the derepression of BES-linked *VSGs* appeared to be much stronger in PF cells than in BF cells. In our previous study, on *Tb*RAP1 depletion, *VSG 3*, *6* and *11* were derepressed up to 8-, 23- and 41-fold, respectively, in BF cells (23), whereas the same genes were derepressed up to 270-, 220- and 260-fold, respectively, in PF cells (Figure 2A).

**Figure 3.**
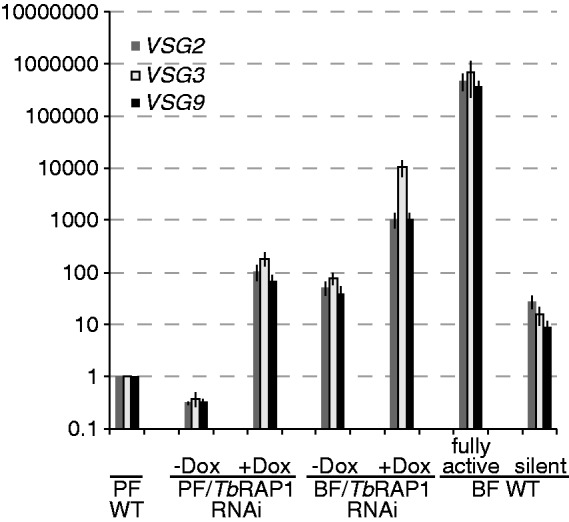
*Tb*RAP1-mediated BES-linked *VSG* silencing is stronger in PF cells than in BF cells. The relative mRNA levels for the same *VSG* genes were estimated by qRT-PCR when the *VSG* is in BF or PF *Tb*RAP1 RNAi cells before (silent) and after (derepressed) *Tb*RAP1 depletion and when the *VSG* is in the active BES in WT BF cells (fully active) or in the silent state in both PF and BF cells (PF WT and silent BF WT, respectively). The mRNA levels at various states were normalized against that in PF WT silent state, which was arbitrarily set to 1. Average values calculated from five independent experiments were presented for each *VSG*. Error bars represent standard deviation. *VSG* derepression in PF cells was analyzed at day 0 and day 2.5 in PRi-pool cells (labeled as PF/*Tb*RAP1 RNAi). At the BF stage, *VSG3* and *VSG9* derepression was analyzed at 0 h and 36 h in Ri-2 cells (expresses VSG2) (23), and *VSG2* derepression was analyzed at the same time points in Ri-9 cells (expresses VSG9) (23), which are labeled as BF/*Tb*RAP1 RNAi. Three BF WT cells were also used: Single Marker (SM) expresses VSG2 (33), pVS3-2/OD1-1 expresses VSG9 (23) and a SM-derived line expresses VSG3. All cell lines are listed in Supplementary Table S1.

To better quantify *VSG* derepression, we used qRT-PCR to carefully compare individual BES-linked *VSG* mRNA levels at the silent state in wild-type (WT) PF and BF cells, at the derepressed state in *Tb*RAP1-depleted PF and BF cells, and at the fully active state in BF cells. The steady state *VSG* mRNA levels under various conditions were normalized against that in PF WT cells, which was arbitrarily set to 1. Three BES-linked *VSGs*—*VSGs 2*, *3* and *9—*were analyzed owing to availability of all necessary cell lines in our laboratory (Supplementary Table S2). We found that first, in WT cells, *VSGs* 2, 3 and 9 mRNA levels were 28-, 16- and 9-fold higher in BF cells than in PF cells, respectively (Figure 3, PF WT and BF WT). This is likely owing to the fact that the *VSG* mRNAs are less stable in PF cells than in BF cells (36). Second, *Tb*RAP1 was essential for *VSG* silencing at both stages. Depletion of *Tb*RAP1 elevated the *VSG2*, *3* and *9* mRNA levels on average 333-, 527- and 217-fold, respectively, in PF cells and on average 22-, 133- and 30-fold, respectively, in BF cells (Figure 3, PF/*Tb*RAP1RNAi −/+ Dox, BF/*Tb*RAP1RNAi −/+ Dox). Third, the derepression of BES-linked *VSGs* resulting from *Tb*RAP1 depletion was significantly stronger in PF cells (217–527-fold) than that in BF cells (22–133-fold). The *P*-values were all <0.002 for *VSG2*, *3* and *9*, in unpaired *t*-tests when comparing changes in BF and PF cells. Therefore, the *Tb*RAP1-mediated BES *VSG* silencing was stronger in PF cells than in BF cells. We noticed that in un-induced PRi-pool cells, the *VSG* mRNA level is ∼3-fold lower than that in PF WT cells (Figure 3), possibly reflecting small variations between different cell populations.

In yeast, longer telomeres confer stronger telomeric silencing (37). However, telomeres in our PF and BF *Tb*RAP1 RNAi cells are of similar length (on average ∼15 kb), suggesting that telomere length is not the reason for different *Tb*RAP1-mediated silencing in BF and PF cells. To rule out the possibility that *Tb*RAP1 might not be equally depleted in BF and PF cells, which could contribute to the differential *VSG*-derepression at the two stages, we examined *Tb*RAP1 protein levels in all *Tb*RAP1 RNAi cells. In Ri-2 and Ri-9 cells, *Tb*RAP1 was depleted as efficiently as described previously (23). At the time points when various *Tb*RAP1 RNAi cells were examined, comparable residual amounts of *Tb*RAP1 were detected (on average, 4% in Ri-2, 14%–11% in Ri-9 and 16% in PRi-pool; Supplementary Figure S6A–C) (23). Therefore, it is unlikely that different levels of *VSG* derepression in BF and PF cells were due to different levels of *Tb*RAP1 depletion. Another possibility is that *Tb*RAP1 was expressed at different levels in BF and PF cells. However, by northern and western analyses, we found that *Tb*RAP1 mRNA and protein levels were approximately the same at the two life cycle stages (Supplementary Figure S6D and E).

Although *Tb*RAP1 is also required for *mVSG* silencing, a higher degree of variation in *mVSG* derepression was observed in independent inductions of *Tb*RAP1 RNAi (Figure 2B and C), and no significant differences were observed between BF and PF cells (*P* > 0.05), except that derepression of *mVSG397* in Ri-BRCT cells is slightly higher than that in PRi-pool cells (*P* = 0.03).

### Depletion of *Tb*RAP1 led to more loosely packed chromatin structures in PF cells

To further investigate whether *Tb*RAP1-mediated *VSG* silencing involves a chromatin remodeling mechanism, we compared the subtelomeric chromatin structure before and after depletion of *Tb*RAP1 using the FAIRE analysis (32), where DNA fragments free of bound proteins are enriched in the final phenol-chloroform-extracted fraction. We used FAIRE to extract subtelomeric BES-linked *VSG* DNAs, whose amount was estimated by quantitative PCR, and the fold change was calculated by dividing the post-*Tb*RAP1 depletion amount by the pre-depletion amount.

**Figure 4.**
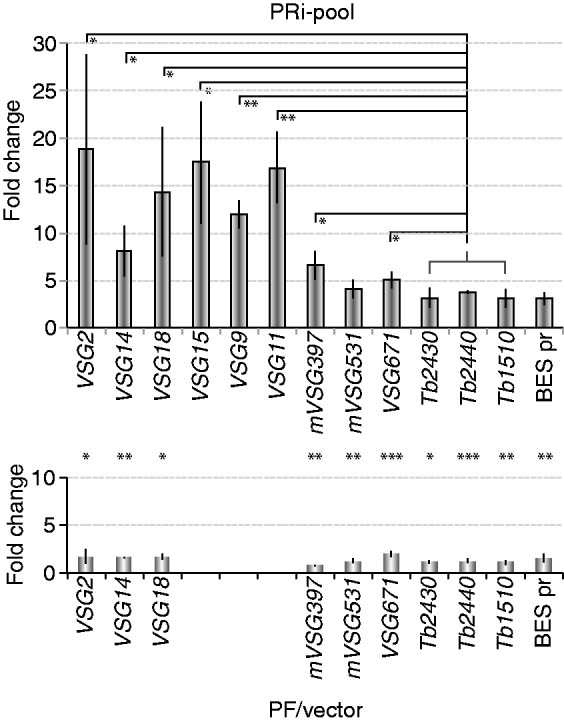
Depletion of *Tb*RAP1 led to loosened chromatin structure, particularly at subtelomeric *VSG* loci, in PF cells. The amount of FAIRE-extracted DNA after depletion of *Tb*RAP1 for 2.5 days was quantified by PCR using primers specific to indicated gene loci and divided by that obtained before depletion of *Tb*RAP1. The average was calculated from at least three independent experiments. Standard deviations are shown as error bars. Top, results from PRi-pool cells; Bottom, results from control cells. The *y*-axes of both diagrams are in the same scale. Within the PRi-pool cells, BES-linked *VSG* FAIRE values were compared with the group of chromosome internal genes, and significant *P*-values are indicated with asterisks. Between PRi-pool and the control cells, the values for each gene were compared and significant differences are marked with asterisks between the two corresponding columns from the two histograms. Control genes are listed using the last 4 digits of their gene ID. *Tb2430*: *Tb927.2.2430*; *Tb2440*: *Tb927.2.2440*; *Tb1510*: *Tb09.211.1510*. *, 0.01 < *P* ≤ 0.05; **, 0.001 < *P* ≤ 0.01, ***, *P* ≤ 0.001.

Depletion of *Tb*RAP1 for 1.5 days did not cause a significant change in FAIRE values in PRi-pool cells (data not shown), probably owing to insufficient *Tb*RAP1 depletion (35% of *Tb*RAP1 protein was left by day 1, Figure 1A). We therefore performed FAIRE at day 2.5 in PRi-pool and found an 8–19-fold increase in BES-linked *VSG* DNA and a 4–7-fold increase in *mVSG* DNA enrichment, which were significantly higher than that in the control cells (Figure 4). To determine whether this effect is telomere-specific, we also examined chromatin structure at several random chromosome-internal gene loci, including *Tb*927.2.2430, *Tb*927.2.2440 and *Tb*09.211.1510. Depletion of *Tb*RAP1 in PF cells led to a 3–4-fold increase in FAIRE-extracted DNAs at these loci, which was mildly but significantly higher than that in the control cells (Figure 4), suggesting that *Tb*RAP1 may have a more global effect on chromatin structure than we anticipated. However, the changes at subtelomeric *VSG* loci were significantly stronger than that at the control loci (*P* < 0.05, Figure 4), indicating that the effect of *Tb*RAP1 on chromatin structure is more prominent at subtelomeric loci. The BES promoter regions that are 40–60 kb upstream of the telomere were similarly affected by *Tb*RAP1 depletion as the chromosome internal regions (Figure 4).

To rule out the possibility that the chromatin structure change resulted from elevated transcription rather than directly from loss of *Tb*RAP1, we examined the *VSG671* locus, which is located at the subtelomere of a minichromosome (Supplementary Figure S3) and not derepressed on *Tb*RAP1 depletion. Depletion of *Tb*RAP1 led to an ∼5-fold increase in FAIRE extracted *VSG671* DNA, which was significantly different from that in control cells (Figure 4), indicating that the *VSG671* chromatin structure was affected by *Tb*RAP1, and that this effect was not due to elevated transcription of *VSG671*.

We subsequently performed the same FAIRE analyses in BF cells in multiple independent *Tb*RAP1 RNAi cell lines (Ri-CT, Ri-BRCT, Ri-2 and Ri-9) at 24 and 36 h after *Tb*RAP1 depletion. A mild increase in the amount of FAIRE-extracted BES-linked, metacyclic and minichromosome *VSG* DNA was visible in all *Tb*RAP1 RNAi and control cells at both time points (Supplementary Figure S7). A similar change was also observed for the chromosome internal genes and the BES promoter loci (1–3-fold) (Supplementary Figure S7). Unpaired *t*-test showed that all *VSG* loci behaved similarly to chromosome internal loci, and all *Tb*RAP1 RNAi cells behaved similarly to the control cells (*P* > 0.05). Therefore, we did not detect significant changes in chromatin structure on depletion of *Tb*RAP1 at the infectious stage.

To rule out the possibility that inefficient depletion of *Tb*RAP1 was the reason for the lack of significant chromatin structure change in BF *Tb*RAP1 RNAi cells, we examined *Tb*RAP1 protein levels by western analysis. At 24 h after *Tb*RAP1 RNAi induction, the *Tb*RAP1 protein level typically reduced to 6 or 26% of the WT level in Ri-CT and Ri-BRCT cells, respectively (Supplementary Figure S6C). By 36 h, Ri-2 and Ri-9 cells had ∼10% or less *Tb*RAP1 left (Supplementary Figure S6A and B). Although in PRi-pool cells, *Tb*RAP1 was typically depleted to ∼16% of the WT level after 2.5 days of induction (Supplementary Figure S6C). Therefore, the different phenotypes we observed in BF and PF cells did not result from unequal levels of *Tb*RAP1 depletion.

**Figure 5.**
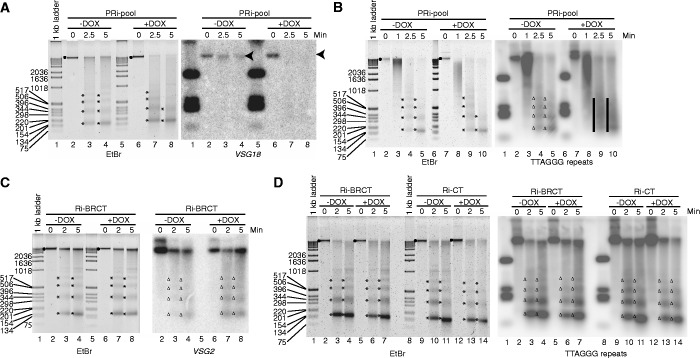
Depletion of *Tb*RAP1 led to chromatin structure changes in PF cells. *Trypanosoma brucei* cells were treated with MNase for an increasing length of time (min) before genomic DNA was isolated, separated on agarose gel, blotted onto nylon membranes and hybridized with specific probes. The PRi-pool cells were treated and hybridized with the *VSG18* (**A**) or the TTAGGG repeat probes (**B**). The Ri-CT and Ri-BRCT cells were treated and hybridized with the *VSG2* (**C**) or the TTAGGG repeat probes (**D**). Black dots and the arrow head represent limiting mobility in ethidium bromide (EtBr)-stained gels and hybridized blots, respectively. Asterisks and triangles represent mono-, di-, tri- and tetra- nucleosomes on EtBr-stained gels and hybridized blots, respectively. Bars represent smeared DNA without distinctive nucleosome laddering pattern.

To further validate our conclusion, we examined the accessibility of chromatin by MNase before and after depletion of *Tb*RAP1. PRi-pool cells were induced for 2.5 days and treated with a fixed amount of MNase for an increasing length of time. Subsequently, the genomic DNA was isolated and separated on agarose gel. Without MNase treatment, nearly all of the genomic DNA was in the limiting mobility size range (Figure 5, EtBr, dots). After 2.5–5 min of digestion with MNase, a portion of the chromatin was digested into mono-, di-, tri-nucleosomes and so forth, and the isolated DNA gave a laddering pattern with the smallest bands of ∼150, 350 and 500 bp (Figure 5, EtBr, asterisks). After depletion of *Tb*RAP1, the chromatin was more accessible to MNase digestion, and less material was left undigested while more DNA was in the mono-nucleosome fraction (Figure 5A, lanes 7 and 8; Figure 5B, lanes 9 and 10). The samples from *Tb*RAP1-depleted cells also appeared more smeared, whereas samples from un-induced cells gave sharper bands (Figure 5A, compare lanes 4 and 8; Figure 5B, compare lanes 4 and 9), indicating that in *Tb*RAP1-depleted cells, DNA was less protected by nucleosomes, resulting in more DNA degradation. Depletion of *Tb*RAP1 altered the MNase digestion pattern mildly in ethidium bromide-stained gels, suggesting that *Tb*RAP1 had an effect on chromatin structure at multiple loci in the genome. On the contrary, adding doxycycline to the control cells did not change the MNase digestion pattern (Supplementary Figure S8E and F).

To examine chromatin structure at specific loci, we carried out Southern blotting using a *VSG18* probe, a *VSG2* probe, an *mVSG397* probe and a TTAGGG repeat probe. In un-induced cells, most of the *VSG18* DNA was not digested by a 5-min treatment of MNase, whereas in *Tb*RAP1-depleted cells, a 2.5-min digestion had already degraded most of the *VSG18* DNA (Figure 5A, *VSG18* hybridized blot, arrow heads). Depletion of *Tb*RAP1 also had similar effects on the *VSG2* (Supplementary Figure S8A), *mVSG397* (Supplementary Figure S8C), and the telomere chromatin (Figure 5B, black bars). Furthermore, we observed the same phenotype in PRi-C2 cells but not the control cells (Supplementary Figure S8B, E and F). The chromatin structure at the ES promotor region appears to be only mildly affected by *Tb*RAP1 depletion (Supplementary Figure S8D), similar to what we observed in FAIRE analysis (Figure 4).

We also performed the MNase digestion in BF cells. A normal laddering pattern was observed in ethidium bromide-stained gels after *Tb*RAP1 was depleted, which was similar to that obtained before *Tb*RAP1 depletion (Figure 5C and D; Supplementary Figure S9A and B). No significant difference was visible between un-induced and induced cells when the chromatin structure at the *VSG18* (Supplementary Figure S9A), *VSG2* (Figure 5C and Supplementary Figure S9B) and the telomere (Figure 5D) loci were examined. Control cells were not affected by doxycycline, either (Supplementary Figure S9C–E). Therefore, no significant change in chromatin structure was detected in BF cells on depletion of *Tb*RAP1.

## DISCUSSION

### TbRAP1 as a key regulator of VSG silencing at different developmental stages

*Trypanosoma brucei* has evolved sophisticated mechanisms to regulate *VSG* expression at different life cycle stages. At the insect stage, silencing both BF *VSGs* and *mVSGs* avoid their degradation by proteases in the midgut of tsetse (9). In BF cells, silencing of all *mVSGs* and all but one BES-linked *VSGs* is critical for successful antigenic variation and a long-term infection. Recent studies have unveiled several factors that affect BES-linked *VSG* silencing: Depletion of *Tb*RAP1 in BF cells led to derepression of all BES-linked *VSGs* for typically several 10-fold (23); deletion of a histone methyltransferase, DOT1b, led to 10-fold higher of BES-linked *VSG* mRNAs in BF cells (20); depletion of *Tb*ORC1, a component of ORC that is involved in DNA replication also led to several fold increase of BES-linked *VSG* mRNA in BF and PF cells (38). In addition, a number of factors including Imitation Switch (ISWI) (17,39), Nucleoplasmin-Like Protein (NLP) (40), Spt16 (18) and DAC3 (19) are necessary for proper regulation of BES promoter activities without affecting *VSG* silencing. It is clear that regulation of BES-linked *VSG* expression involves multiple mechanisms (14,15,17–20,23,36). On the contrary, regulation of *mVSG* expression appears to be entirely at the transcription initiation level (41,42), which is unusual in *T. brucei*, where posttranscriptional regulation is predominant (43). However, *mVSG* silencing is poorly understood except that *Tb*ORC1, when depleted, led to *mVSG* derepression in PF cells (44).

In this study, we found that depletion of *Tb*RAP1 in PF cells has a similar effect as in BF cells—BES-linked *VSG* mRNA levels were increased several 10- to several 100-fold. In addition, *Tb*RAP1 is also required for *mVSG* silencing at both BF and PF stages. Therefore, *Tb*RAP1 is required for silencing both BES-linked and m*VSGs*, the two types of *VSG* that can be expressed under normal physiological conditions, and *Tb*RAP1 apparently has a much stronger effect on *VSG* silencing than other known *VSG* regulation factors.

### Regulation of VSG expression/silencing at BF and PF stages

*VSGs* are expressed in a strictly monoallelic manner in BF cells and are silenced at the PF stage. In this study, using qRT-PCR analysis, we showed that the steady state mRNA levels of several BES-linked *VSGs* are much lower in PF cells than that in BF cells. A previous study indicated that the 3′ untranslated region of the *VSG* gene renders its mRNA less stable in PF cells (36), which most likely is the reason for a lower *VSG* mRNA level in PF cells than in BF cells.

Regardless of the steady–state levels of *VSG* mRNA in WT BF and PF cells, the changes in *VSG* mRNA levels induced by depletion of *Tb*RAP1 reflect the silencing effect of *Tb*RAP1. The strength of *Tb*RAP1-mediated silencing at the BES-linked *VSG* loci is significantly stronger in PF cells than in BF cells. Although we do not have all the proper cell lines to carry out a comprehensive comparison for all BES-linked *VSGs*, comparison between our current and previous results suggest that most BES-linked *VSGs* are derepressed at a higher level in PF cells (several 10- to several 100-fold) than in BF cells (several to several 10-fold) (23). However, we noticed that different BES-linked *VSGs* were derepressed to different levels on depletion of *Tb*RAP1. It is therefore possible that for some *VSGs*, the derepression levels are at more comparable levels between BF and PF than other *VSGs*. Importantly, depletion of *Tb*RAP1 affects chromatin structure significantly in PF cells, but not in BF cells, suggesting that modulating chromatin structure in PF cells is a mechanism of *Tb*RAP1-mediated *VSG* silencing.

### TbRAP1 modulates chromatin structure in PF cells

A number of recent studies have shown that modulation of chromatin structure is important for regulation of BES expression in BF cells. First, chromatin structures of the active and silent BESs are dramatically different (21,22). Second, several proteins with known functions in chromatin remodeling, including ISWI (17,39), DAC3 (19), Spt16 (18) and DOT1b (20) have been shown to play important roles in regulation of BES promoter activity. In this study, we showed that depletion of *Tb*RAP1 led to a more loosely packed chromatin structure at all genomic loci tested and more significantly at subtelomeric *VSG* regions in PF cells, suggesting that *Tb*RAP1-mediated *VSG* silencing involves modulation of chromatin structure.

Depletion of *Tb*RAP1 in PF cells led to a striking change in the chromatin structure at subtelomeric *VSG* loci, including the minichromosome *VSG671* locus, whereas the *VSG671* mRNA level was not affected, indicating that the change in chromatin structure is not a consequence of elevated gene transcription, and that modulation of chromatin structure is a primary effect of *Tb*RAP1. *VSG671* was not derepressed, presumably because it lacks a functional upstream promoter. However, for BES-linked *VSGs* and *mVSGs*, a loosened chromatin structure most likely is the cause or one of the reasons of their derepression. We noticed that changes in FAIRE values at some BES-linked *VSG* loci (including *VSGs 2*, *18*, *15*, *9* and *11*) are slightly higher than that at the *VSG671* locus (*P* < 0.05). As all BES-linked *VSGs* were strongly derepressed on depletion of *Tb*RAP1, it is possible that the elevated transcription of BES-linked *VSGs* may induce a further opening of the chromatin structure in addition to the initial effect resulting from the depletion of *Tb*RAP1.

At the insect stage, FAIRE and MNase digestion results indicated that *Tb*RAP1 depletion affects chromatin structure mildly at many loci throughout the genome, which is much broader than we previously anticipated. In both yeast and mammalian cells, RAP1 homologs have been found to locate at non-telomeric regions and act as transcription regulators (45–49), and yeast RAP1 has been found to help determine genome-wide chromatin structure (50). We previously observed that some *Tb*RAP1 appears to localize at loci other than telomeres (23). Therefore, *Tb*RAP1 may have a conserved function as its yeast and mammalian homologs in determination of genome-wide chromatin structure.

To our surprise, we did not detect significant chromatin structure changes in BF cells on *Tb*RAP1 depletion. Our observations suggest that the significantly more severe chromatin structure change and the significantly stronger gene derepression at BESs in PF cells than those in BF cells are linked. Currently, we favor several possible explanations (not necessarily mutually exclusive) for the different phenotypes observed in BF and PF cells. First, the BES chromatin may have different structures in BF and PF cells, and *Tb*RAP1 may be the responsible factor for this difference. An earlier work by Navarro *et al.* (51) found that T7 polymerase-mediated transcription from a chromosomally integrated T7 promoter is repressed along the entire length of the BES in PF cells, but not in BF cells. Therefore, the chromatin structure at the silent BES appears to be more tightly packed in PF cells. It is possible that *Tb*RAP1 is the factor that maintains a more closed BES structure in PF cells. Second, as BF cells undergo antigenic variation and silent BESs need to be ready to switch to the active state, their silent state is likely metastable, similar to what is often observed for telomeric silencing in yeast (24). Therefore, the BES may switch between the open and closed states more frequently in BF cells than in PF cells. In this case, *Tb*RAP1 may still be involved in determination of chromatin structures in BF cells, but our techniques are not sensitive enough to detect a significant change owing to the fast switching status of the BES. Another possibility is that more factors are involved in modulating BES chromatin structure and BES silencing independent of *Tb*RAP1 at the BF stage. Therefore, depletion of *Tb*RAP1 in BF cells may still affect the chromatin structure, but a lesser degree of change or no quantifiable differences can be detected. Glover and Horn showed that an rRNA promoter-driven reporter gene targeted 5 kb from the telomere in a silent BES was expressed in PF cells, but not in BF cells (52), suggesting that additional factors are involved in silencing BESs in BF cells. A recent study suggested that depletion of histone H1 led to a chromatin opening at multiple genomic loci only in BF cells, but not in PF cells (53), which is also consistent with this hypothesis.

In this study, we further demonstrated that *Tb*RAP1 is a key regulator of *VSG* expression in *T. brucei* cells, and that *VSG* silencing appears to involve chromatin structure. In PF cells, *Tb*RAP1 helps to determine the chromatin structure, particularly at the subtelomeric BES loci, which presumably contributes to *Tb*RAP1-mediated silencing. The effect of *Tb*RAP1 on chromatin structure also appears to be more global than we anticipated.

## SUPPLEMENTARY DATA

Supplementary Data are available at NAR Online: Supplementary Tables 1 and 2, Supplementary Figures 1–9 and Supplementary Methods.

## FUNDING

National Institutes of Heath (NIH) [AI066095 to B.L.]. Funding for open access charge: [NIH R01].

*Conflict of interest statement.* None declared.

## Supplementary Material

Supplementary Data

## References

[1] Cross GAM (1975). Identification, purification and properties of clone-specific glycoprotein antigens constituting the surface coat of *Trypanosoma brucei*. Parasitology.

[2] Reinitz DM, Mansfield JM (1990). T-cell-independent and T-cell-dependent B-cell responses to exposed variant surface glycoprotein epitopes in trypanosome-infected mice. Infect. Immun..

[3] Barry JD, McCulloch R (2001). Antigenic variation in trypanosomes: enhanced phenotypic variation in a eukaryotic parasite. Adv. Parasitol..

[4] Berriman M, Ghedin E, Hertz-Fowler C, Blandin G, Renauld H, Bartholomeu DC, Lennard NJ, Caler E, Hamlin NE, Haas B (2005). The genome of the African trypanosome *Trypanosoma brucei*. Science.

[5] Gunzl A, Bruderer T, Laufer G, Schimanski B, Tu LC, Chung HM, Lee PT, Lee MG (2003). RNA polymerase I transcribes procyclin genes and variant surface glycoprotein gene expression sites in *Trypanosoma brucei*. Eukaryot. Cell.

[6] de Lange T, Borst P (1982). Genomic environment of the expression-linked extra copies of genes for surface antigens of *Trypanosoma brucei* resembles the end of a chromosome. Nature.

[7] Navarro M, Cross GAM (1996). DNA rearrangements associated with multiple consecutive directed antigenic switches in *Trypanosoma brucei*. Mol. Cell. Biol..

[8] Hertz-Fowler C, Figueiredo LM, Quail MA, Becker M, Jackson A, Bason N, Brooks K, Churcher C, Fahkro S, Goodhead I (2008). Telomeric expression sites are highly conserved in *Trypanosoma brucei*. PLoS One.

[9] Gruszynski AE, van Deursen FJ, Albareda MC, Best A, Chaudhary K, Cliffe LJ, del Rio L, Dunn JD, Ellis L, Evans KJ (2006). Regulation of surface coat exchange by differentiating African trypanosomes. Mol. Biochem. Parasitol..

[10] Tetley L, Turner CMR, Barry JD, Crowe JS, Vickerman K (1987). Onset of expression of the variant surface glycoproteins of *Trypanosoma brucei* in the tsetse fly studied using immunoelectron microscopy. J. Cell Sci..

[11] Barry JD, Graham SV, Fotheringham M, Graham VS, Kobryn K, Wymer B (1998). *VSG* gene control and infectivity strategy of metacyclic stage *Trypanosoma brucei*. Mol. Biochem. Parasitol..

[12] Ginger ML, Blundell PA, Lewis AM, Browitt A, Gunzl A, Barry JD (2002). Ex vivo and in vitro identification of a consensus promoter for *VSG* genes expressed by metacyclic–stage trypanosomes in the tsetse fly. Eukaryot. Cell.

[13] Alarcon CM, Son HJ, Hall T, Donelson JE (1994). A monocistronic transcript for a trypanosome variant surface glycoprotein. Mol. Cell. Biol..

[14] Navarro M, Gull K (2001). A pol I transcriptional body associated with *VSG* mono–allelic expression in *Trypanosoma brucei*. Nature.

[15] Vanhamme L, Poelvoorde P, Pays A, Tebabi P, Van Xong H, Pays E (2000). Differential RNA elongation controls the variant surface glycoprotein gene expression sites of *Trypanosoma brucei*. Mol. Microbiol..

[16] Ansorge I, Steverding D, Melville S, Hartmann C, Clayton C (1999). Transcription of ‘inactive' expression sites in African trypanosomes leads to expression of multiple transferrin receptor RNAs in bloodstream forms. Mol. Biochem. Parasitol..

[17] Hughes K, Wand M, Foulston L, Young R, Harley K, Terry S, Ersfeld K, Rudenko G (2007). A novel ISWI is involved in *VSG* expression site downregulation in African trypanosomes. EMBO J..

[18] Denninger V, Fullbrook A, Bessat M, Ersfeld K, Rudenko G (2010). The FACT subunit TbSpt16 is involved in cell cycle specific control of *VSG* expression sites in *Trypanosoma brucei*. Mol. Microbiol..

[19] Wang QP, Kawahara T, Horn D (2010). Histone deacetylases play distinct roles in telomeric *VSG* expression site silencing in African trypanosomes. Mol. Microbiol..

[20] Figueiredo LM, Janzen CJ, Cross GAM (2008). A histone methyltransferase modulates antigenic variation in African trypanosomes. PLoS Biol..

[21] Figueiredo LM, Cross GAM (2010). Nucleosomes are depleted at the *VSG* expression site transcribed by RNA polymerase I in African trypanosomes. Eukaryot. Cell.

[22] Stanne TM, Rudenko G (2010). Active *VSG* expression sites in *Trypanosoma brucei* are depleted of nucleosomes. Eukaryot. Cell.

[23] Yang X, Figueiredo LM, Espinal A, Okubo E, Li B (2009). RAP1 is essential for silencing telomeric variant surface glycoprotein genes in *Trypanosoma brucei*. Cell.

[24] Gottschling DE, Aparicio OM, Billington BL, Zakian VA (1990). Position effect at *S. cerevisiae* telomeres: reversible repression of pol II transcription. Cell.

[25] Baur JA, Zou Y, Shay JW, Wright WE (2001). Telomere position effect in human cells. Science.

[26] Pedram M, Sprung CN, Gao Q, Lo AW, Reynolds GE, Murnane JP (2006). Telomere position effect and silencing of transgenes near telomeres in the mouse. Mol. Cell. Biol..

[27] Horn D, Cross GAM (1995). A developmentally regulated position effect at a telomeric locus in *Trypanosoma brucei*. Cell.

[28] Freitas-Junior LH, Hernandez-Rivas R, Ralph SA, Montiel-Condado D, Ruvalcaba-Salazar OK, Rojas-Meza AP, Mancio-Silva L, Leal-Silvestre RJ, Gontijo AM, Shorte S (2005). Telomeric heterochromatin propagation and histone acetylation control mutually exclusive expression of antigenic variation genes in malaria parasites. Cell.

[29] Duraisingh MT, Voss TS, Marty AJ, Duffy MF, Good RT, Thompson JK, Freitas-Junior LH, Scherf A, Crabb BS, Cowman AF (2005). Heterochromatin silencing and locus repositioning linked to regulation of virulence genes in *Plasmodium falciparum*. Cell.

[30] Munoz-Jordan JL, Cross GAM, de Lange T, Griffith JD (2001). t-loops at trypanosome telomeres. EMBO J..

[31] Li B, Espinal A, Cross GAM (2005). Trypanosome telomeres are protected by a homologue of mammalian TRF2. Mol. Cell. Biol..

[32] Giresi PG, Lieb JD (2009). Isolation of active regulatory elements from eukaryotic chromatin using FAIRE (Formaldehyde Assisted Isolation of Regulatory Elements). Methods.

[33] Wirtz E, Leal S, Ochatt C, Cross GAM (1999). A tightly regulated inducible expression system for dominant negative approaches in *Trypanosoma brucei*. Mol. Biochem. Parasitol..

[34] Kolev NG, Ramey-Butler K, Cross GAM, Ullu E, Tschudi C (2012). Developmental progression to infectivity in *Trypanosoma brucei* triggered by an RNA-binding protein. Science.

[35] Wang Z, Morris JC, Drew ME, Englund PT (2000). Inhibition of *Trypanosoma brucei* gene expression by RNA interference using an integratable vector with opposing T7 promoters. J. Biol. Chem..

[36] Berberof M, Vanhamme L, Tebabi P, Pays A, Jefferies D, Welburn S, Pays E (1995). The 3'-terminal region of the mRNAs for *VSG* and procyclin can confer stage specificity to gene expression in *Trypanosoma brucei*. EMBO J.

[37] Kyrion G, Liu K, Liu C, Lustig AJ (1993). RAP1 and telomere structure regulate telomere position effects in *Saccharomyces cerevisiae*. Genes Dev..

[38] Benmerzouga I, Concepcion-Acevedo J, Kim HS, Vandoros AV, Cross GAM, Klingbeil MM, Li B (2013). *Trypanosoma brucei* Orc1 is essential for nuclear DNA replication and affects both *VSG* silencing and *VSG* switching. Mol. Microbiol..

[39] Stanne TM, Kushwaha M, Wand M, Taylor JE, Rudenko G (2011). TbISWI regulates multiple polymerase I (Pol I)-transcribed loci and is present at Pol II transcription boundaries in *Trypanosoma brucei*. Eukaryot. Cell.

[40] Narayanan MS, Kushwaha M, Ersfeld K, Fullbrook A, Stanne TM, Rudenko G (2011). NLP is a novel transcription regulator involved in *VSG* expression site control in *Trypanosoma brucei*. Nucleic Acids Res..

[41] Graham SV, Barry JD (1995). Transcriptional regulation of metacyclic variant surface glycoprotein gene expression during the life cycle of *Trypanosoma brucei*. Mol. Cell. Biol..

[42] Pedram M, Donelson JE (1999). The anatomy and transcription of a monocistronic expression site for a metacyclic variant surface glycoprotein gene in *Trypanosoma brucei*. J. Biol. Chem..

[43] Clayton C, Shapira M (2007). Post-transcriptional regulation of gene expression in trypanosomes and leishmanias. Mol. Biochem. Parasitol..

[44] Tiengwe C, Marcello L, Farr H, Dickens N, Kelly S, Swiderski M, Vaughan D, Gull K, Barry JD, Bell SD (2012). Genome-wide analysis reveals extensive functional interaction between DNA replication initiation and transcription in the genome of *Trypanosoma brucei*. Cell Rep..

[45] Shore D (1994). RAP1: a protean regulator in yeast. Trends Genet..

[46] Yarragudi A, Parfrey LW, Morse RH (2007). Genome-wide analysis of transcriptional dependence and probable target sites for Abf1 and Rap1 in *Saccharomyces cerevisiae*. Nucleic Acids Res..

[47] Yang D, Xiong Y, Kim H, He Q, Li Y, Chen R, Songyang Z (2011). Human telomeric proteins occupy selective interstitial sites. Cell Res..

[48] Teo H, Ghosh S, Luesch H, Ghosh A, Wong ET, Malik N, Orth A, de Jesus P, Perry AS, Oliver JD (2010). Telomere-independent Rap1 is an IKK adaptor and regulates NF-kappaB-dependent gene expression. Nat. Cell. Biol..

[49] Martinez P, Thanasoula M, Carlos AR, Gomez-Lopez G, Tejera AM, Schoeftner S, Dominguez O, Pisano DG, Tarsounas M, Blasco MA (2010). Mammalian Rap1 controls telomere function and gene expression through binding to telomeric and extratelomeric sites. Nat. Cell. Biol..

[50] Ganapathi M, Palumbo MJ, Ansari SA, He Q, Tsui K, Nislow C, Morse RH (2011). Extensive role of the general regulatory factors, Abf1 and Rap1, in determining genome-wide chromatin structure in budding yeast. Nucleic Acids Res..

[51] Navarro M, Cross GAM, Wirtz E (1999). *Trypanosoma brucei* variant surface glycoprotein regulation involves coupled activation/inactivation and chromatin remodeling of expression sites. EMBO J..

[52] Glover L, Horn D (2006). Repression of polymerase I-mediated gene expression at *Trypanosoma brucei* telomeres. EMBO Rep..

[53] Povelones ML, Gluenz E, Dembek M, Gull K, Rudenko G (2012). Histone H1 Plays a Role in Heterochromatin Formation and *VSG* Expression Site Silencing in *Trypanosoma brucei*. PLoS Pathog..

