# Supplementary material for: Silencing subtelomeric VSGs by Trypanosoma brucei RAP1 at the insect stage involves chromatin structure changes
Source: Nucleic Acids Res. 2013 Jun 26;41(16):7673–82. doi: 10.1093/nar/gkt562 (PMC3763547; doi:10.1093/nar/gkt562)
Supplement: Supplementary Data [file supp_41_16_7673__index.html]

Silencing subtelomeric VSGs by Trypanosoma brucei RAP1 at the insect stage involves chromatin structure changes — Silencing subtelomeric VSGs by Trypanosoma brucei RAP1 at the insect stage involves chromatin structure changes — Supplementary Data 

# Silencing subtelomeric *VSGs* by *Trypanosoma brucei* RAP1 at the insect stage involves chromatin structure changes

## 

files

**Files in this Data Supplement:**

- Supplementary Data - pdf file
