# Supplementary material for: Silencing subtelomeric VSGs by Trypanosoma brucei RAP1 at the insect stage involves chromatin structure changes
Source: Nucleic Acids Res. 2013 Jun 26;41(16):7673–82. doi: 10.1093/nar/gkt562 (PMC3763547; doi:10.1093/nar/gkt562)
Supplement: Supplementary Data [file supp_gkt562_nar-00518-x-2013-File007.pdf]

**Supplementary Materials**

**Supplementary Figure Legends**

Supplementary figure 1. Depletion of *Tb*RAP1 led to growth arrest and derepression of BES-linked *VSGs* in PF cells. (A–C) Growth curves of three independent PF/*Tb*RAP1 RNAi clones, PRi-C1 (A), PRi-C2 (B), and PRi-B2 (C). Average Population Doublings (PDs) versus days with (+Dox) or without (-Dox) induction were plotted from three independent experiments. Error bars represent standard deviation. As the RNAi construct is targeted to one of many rDNA spacer loci with various expressing levels, ineffective RNAi is often due to the low expression of the RNAi construct in specific clones. Therefore, clone C1 and B2 are not studied further in case *Tb*RAP1 depletion is not efficient enough in these cells. (D) Derepression of two BES-linked *VSGs* upon *Tb*RAP1 depletion in PRi-C2. Steady state mRNA levels of the tested genes at days 0, 2, and 3 after induction of *Tb*RAP1 RNAi were estimated by qRT-PCR and normalized against those before induction. Fold change in the mRNA levels is plotted. Day 0 value was set to "1" after normalization but not shown. (E) Western analysis of various protein levels in the PRi-pool (left) and the control cells with the empty RNAi vector (right). (F) Western analyses of *Tb*RAP1 and EF-2 protein levels in PRi-C2 cells at the indicated time points after induction of *Tb*RAP1 RNAi.

Supplementary figure 2. Flow cytometry analysis of the PRi-pool (A) and Ri-CT (B) cells. Results from two independent analyses in each cell line are shown. Cells were analyzed at Days 0, 1, 2, and 3 (A) or 0, 18, 24, and 36 hrs (B) after induction of *Tb*RAP1 RNAi. (C) Quantification of cell populations at different cell cycle stages before (0 hr) and after (18, 24, and 36 hrs) depletion of *Tb*RAP1 in Ri-CT cells. Unpaired t-tests were performed to compare 18, 24, and 36 hr values with the 0 hr value. *, $0.01<P\leq 0.05$; **, $0.001<P\leq 0.01$.

Supplementary figure 3. Southern analysis to confirm the presence of *VSG671* on a minichromosome in various *Tb*RAP1 RNAi cell lines. DNA plugs were prepared from BF and PF *Tb*RAP1 RNAi cells and separated by Pulsed Field Gel

Electrophoresis under the conditions to separate intact *T. brucei* chromosomes (31). Left, ethidium bromide-stained gel. The blue arrow marks the minichromosome species. Right, blotted DNA hybridized with the *VSG671* probe. Marker, *H. wingei* chromosomal DNA (1 – 3.1 Mb) (Bio-Rad).

Supplementary figure 4. Depletion of *Tb*RAP1 resulted in *mVSG* derepression in Ri-BRCT cells (C) but no *VSG* derepression in either PF (A & B) or BF (D) vector control cells. Steady state mRNA levels for BES-linked *VSGs 2, 3, 6, 11, 16,* metacyclic *VSGs 397*, *531*, *639*, *653*, and control genes at days 0, 2, and 3 (PF) or at 0, 24, and 36 hrs (BF) after adding doxycycline in indicated cells were estimated using qRT-PCR and normalized against that before induction. Day 0 value is set to 1 but not shown. The fold change in mRNA levels for three independent induction experiments was shown.

Supplementary figure 5. Induction of *Tb*RAP1 RNAi led to growth defects and derepression of BES-linked *VSGs* in Ri-CT and Ri-BRCT cells. (A) A representative growth curve of the two *Tb*RAP1 RNAi cell lines in the presence (+Dox) or the absence (-Dox) of doxycycline. qRT-PCR results of several induction experiments in Ri-CT (B) and Ri-BRCT (C) cells were shown. Steady state mRNA levels of several BES-linked *VSGs* were estimated before (0 hr) and after (24 hr) induction of *Tb*RAP1 RNAi. The fold change in mRNA levels was calculated and shown for each tested *VSG* gene.

Supplementary figure 6. (A–C) *Tb*RAP1 depletion is similarly efficient in different *Tb*RAP1 RNAi cells. Representative western analyses of the *Tb*RAP1 (tagged with Ty1 in Ri-2 and Ri-9 cells (23)) protein levels at different time points after induction of *Tb*RAP1 RNAi in Ri-2 (A), Ri-9 (B), Ri-CT, Ri-BRCT, and PRi-pool cells (C). Numbers beneath the blot indicate the average relative amount of the *Tb*RAP1 protein level calculated from 2 (Ri-2 and Ri-9) or 3 (Ri-CT, Ri-BRCT, and PRi-pool) independent experiments. (D–E) *Tb*RAP1 is expressed at the same level in BF and PF cells. (D) Northern analysis of *Tb*RAP1 mRNA at the BF

and PF stages (top). Numbers beneath the blot indicate the relative ratio of the *Tb*RAP1 mRNA level. rRNAs are shown as loading control (bottom). (E) Western analysis of the *Tb*RAP1 protein at the two stages. Rabbit anti-*Tb*RAP1 antibody, goat antibody against EF-2 (Santa Cruz Biotechnology Inc.), and TAT-1 anti-tubulin antibody (a generous gift from Dr. Keith Gull) were used.

Supplementary figure 7. Depletion of *Tb*RAP1 does not affect chromatin structure significantly in BF cells. The amount of FAIRE-extracted DNA after depletion of *Tb*RAP1 for 24 hrs (in Ri-CT, Ri-BRCT, and vector control cells) or 36 hrs (in Ri-2 and Ri-9 cells) was quantified by PCR using primers specific to various *VSGs* or control genes and divided by that obtained before RNAi induction. Averages were calculated from at least three independent experiments, and standard deviations are shown as error bars. Control genes are listed using the last 4 digits of their gene ID. TB0330: Tb11.0330; Tb2430: Tb927.2.2430; Tb2440: Tb927.2.2440; Tb1510: Tb09.211.1510.

Supplementary Figure 8. Depletion of *Tb*RAP1 led to chromatin structure changes in PF cells. PF cells were induced for 2.5 days and BF cells for 24 hrs. *T. brucei* nuclei were treated with 1 unit of MNase (Worthington Biochemicals) for an increasing length of time (min) before genomic DNA was isolated and separated on agarose gel, blotted onto Nylon membranes, and hybridized with specific probes. The cells and probes used in different panels are as follows: (A) PRi-pool, *VSG2*; (B) PRi-C2, *VSG18*; (C) PRi-pool, *mVSG397;* (D) PRi-pool, BES promoter; (E) PF/vector, *VSG2*; (F) PF/vector, *VSG18.*

Supplementary Figure 9. No significant chromatin structure changes were detected in BF cells upon depletion of *Tb*RAP1. BF cells were induced for 24 hrs. MNase digestion was performed as describe in Supplementary Figure 8. The cells and probes used in different panels are as follows: (A) Ri-BRCT, *VSG18*;

(B) Ri-CT, *VSG2*; (C) BF/vector, *VSG2*; (D) BF/vector, TTAGGG repeats. (E) BF/vector, *VSG18*.

**Supplementary Table Legends**

Supplementary Table 1. *VSG* nomenclature

For all BES-linked *VSGs* tested in this work, their aliases (used in various previous publications), their previous *MITat* names, and the BESs in which they reside in the 427 strain (8) are listed.

Supplementary Table 2. *Tb*RAP1 RNAi and control cell lines used in this study. Cell line names, their parents or common names, their life cycle stages, transfected vectors for establishing the cell line, expected active *VSGs*, several silent *VSGs* tested in this study, and corresponding references are listed.

**Supplementary Materials and Methods**

*Plasmids and cell lines*

pZJMβ-*Tb*RAP1-CT was generated by inserting the *Tb*RAP1 C-terminus (aa 556 to 855) into the pZJMβ vector (35) at XbaI and XhoI sites. pZJMβ-*Tb*RAP1-BRCT was generated by inserting the *Tb*RAP1 BRCT domain into pZJMβ. BLAST search using these *Tb*RAP1 fragments as queries against the *T. brucei* genome indicated that they are unique. Transfection of pZJMβ-*Tb*RAP1-CT into 29-13 cells (33) resulted in clones PRi-C1, C2, B2, and the PRi-pool (selected without a limiting dilution cloning process after transfection). Ri-BRCT and Ri-CT were established by transfecting SM cells (33) with pZJMβ-*Tb*RAP1-BRCT and pZJMβ-*Tb*RAP1-CT, respectively.

*Pulsed Field Gel Electrophoresis*

DNA plugs were prepared as described in (31). Intact chromosomes were separated on a CHEF DRII (Bio-Rad) for 120 hrs at 14ºC with 700–1500s pulses and a voltage of 2.5 V/cm.

Pandya et al.
Supplementary Figure 1

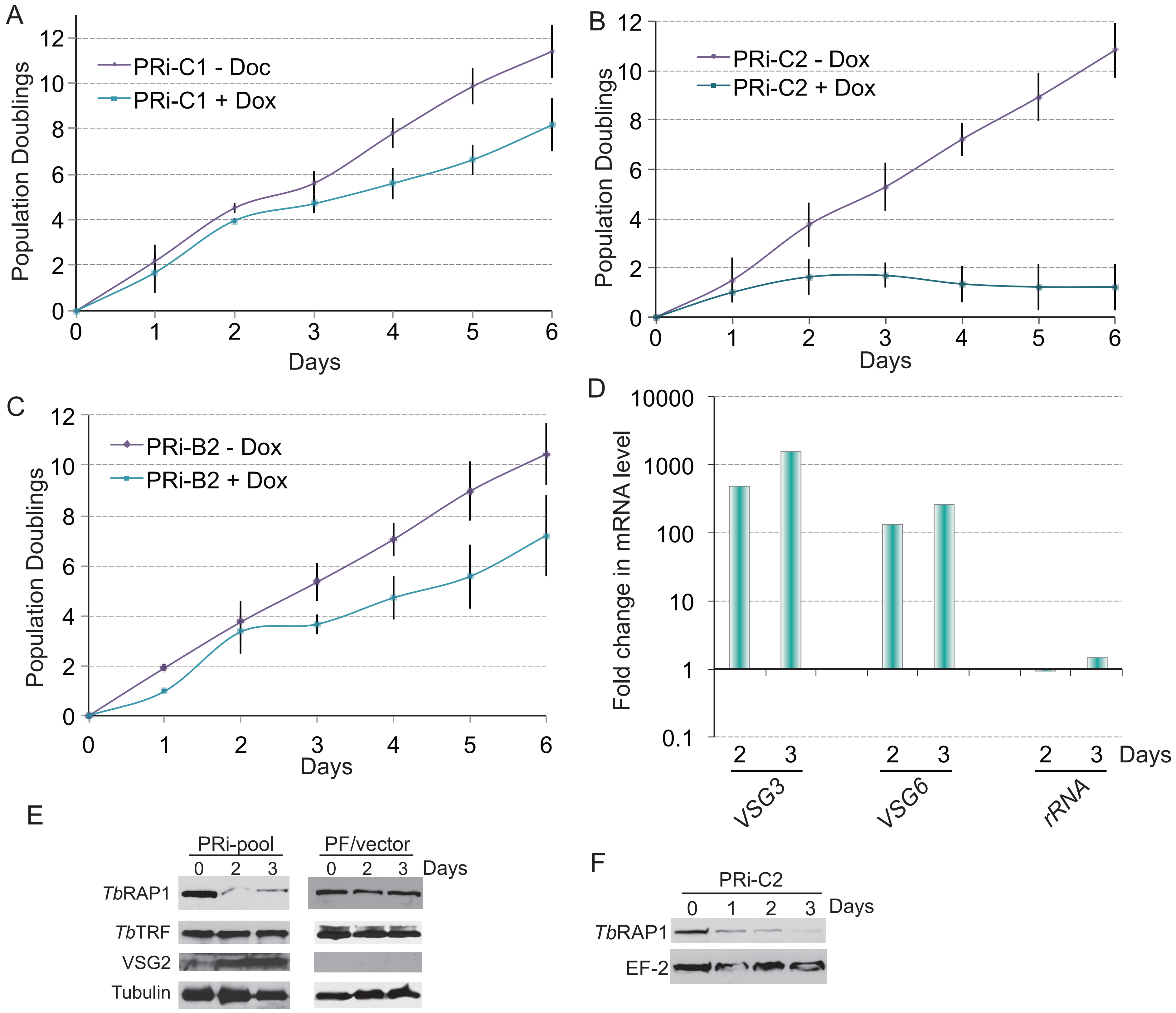

Pandya et al.
Supplementary Figure 2

A

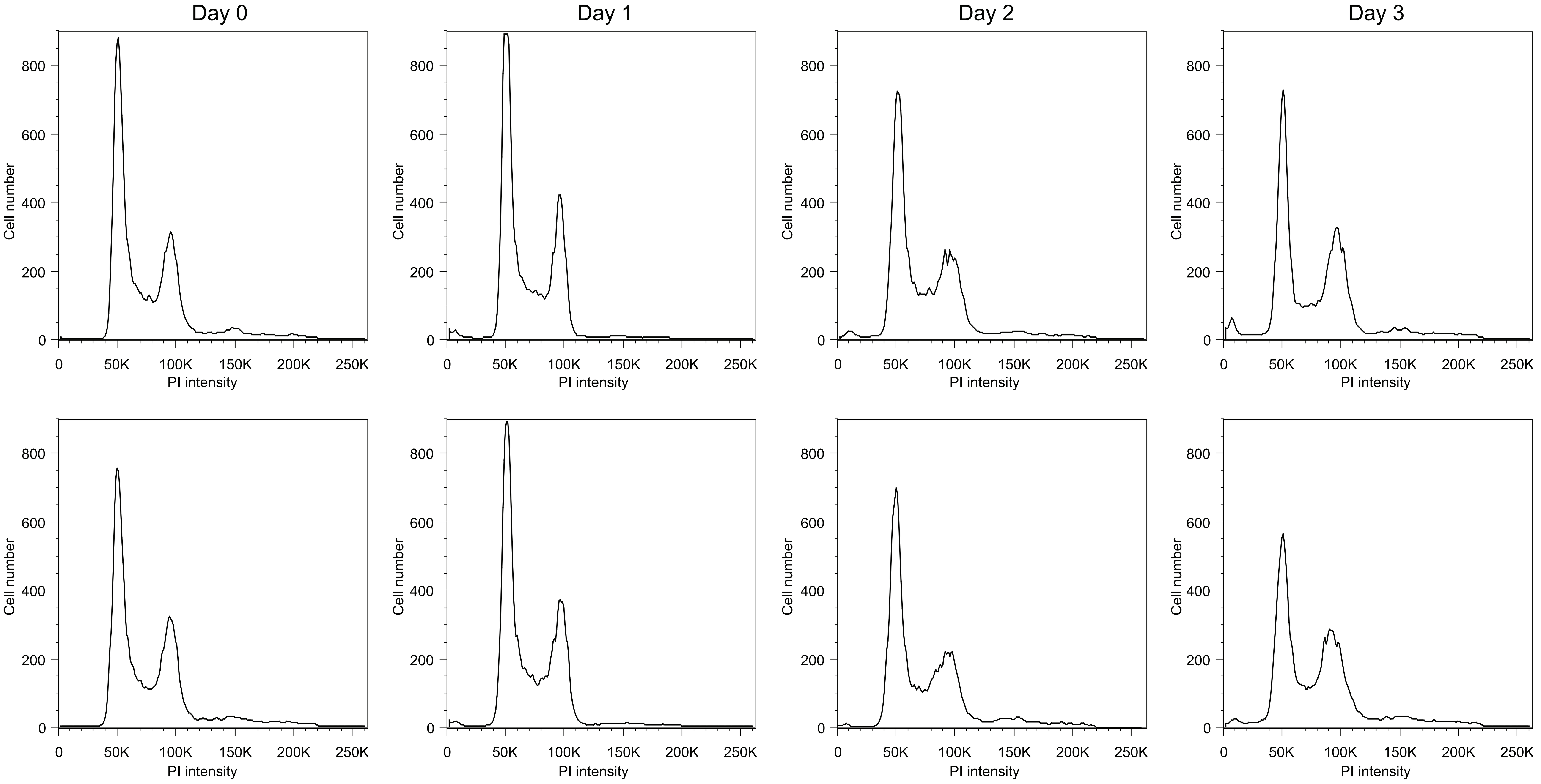


B

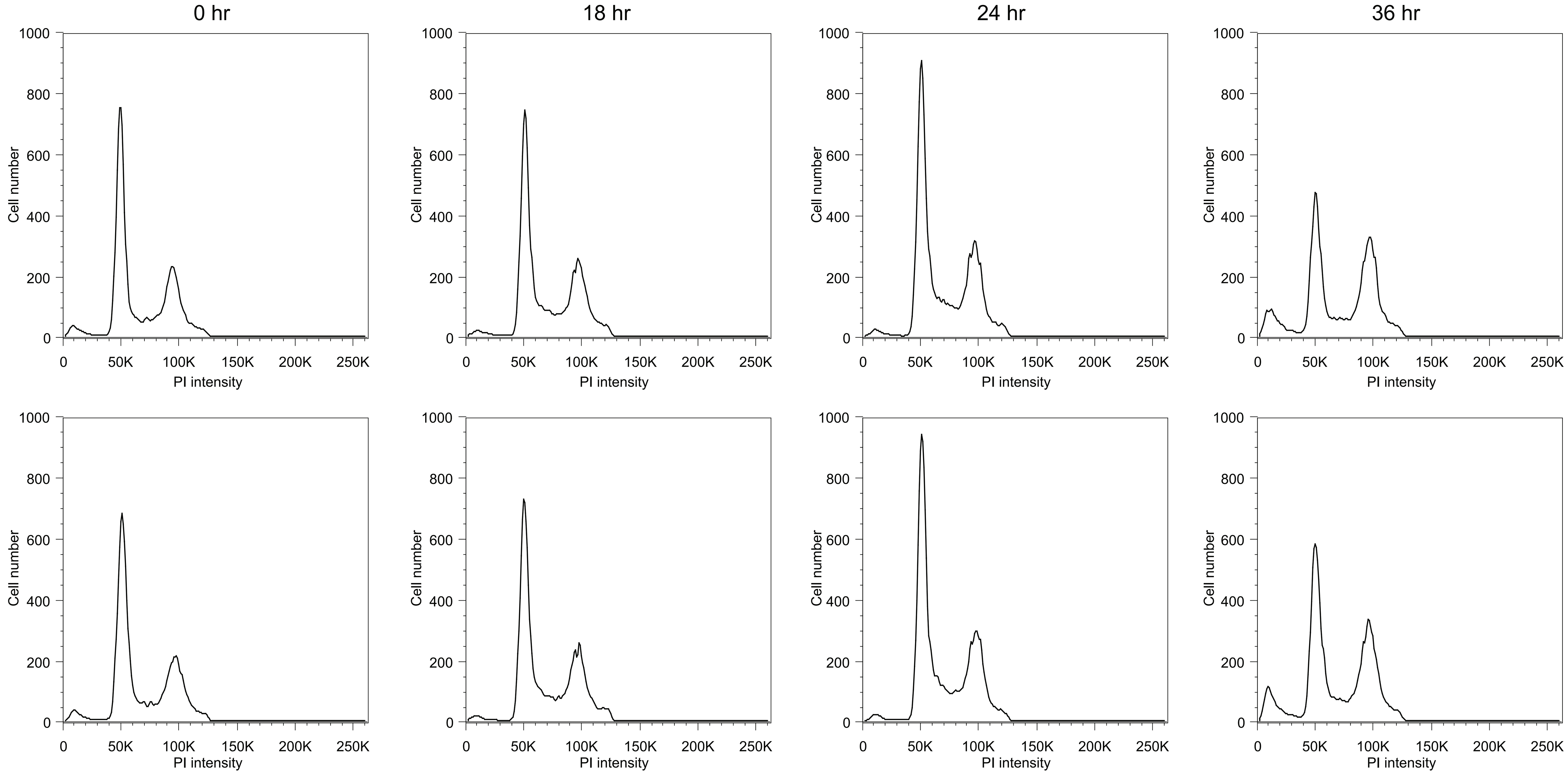


C

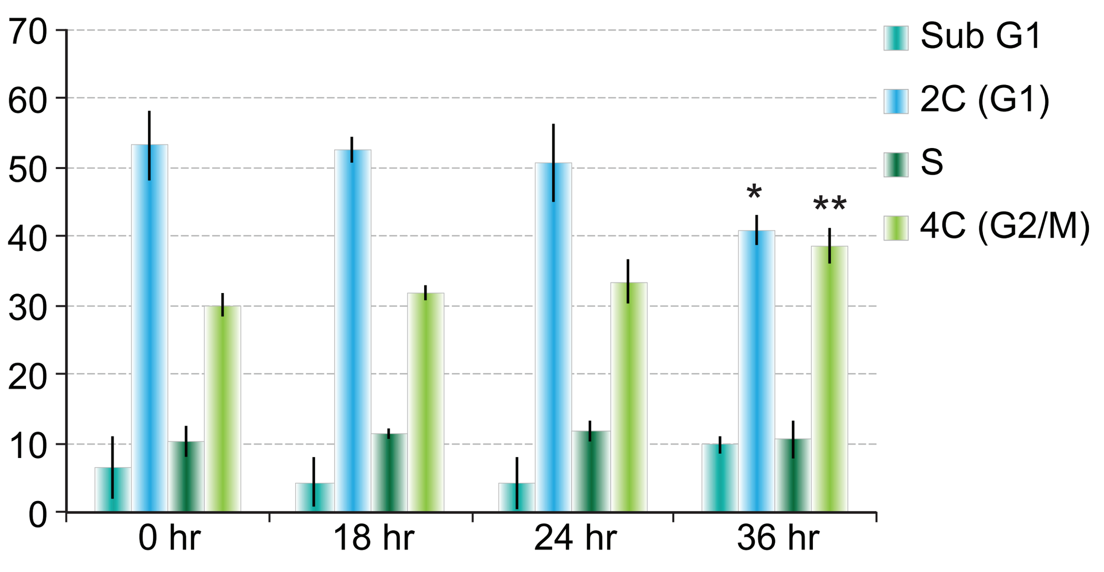

Pandya et al.
Supplementary Figure 3

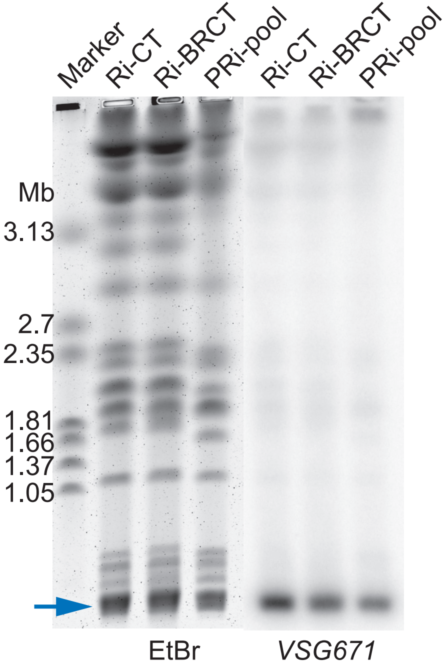

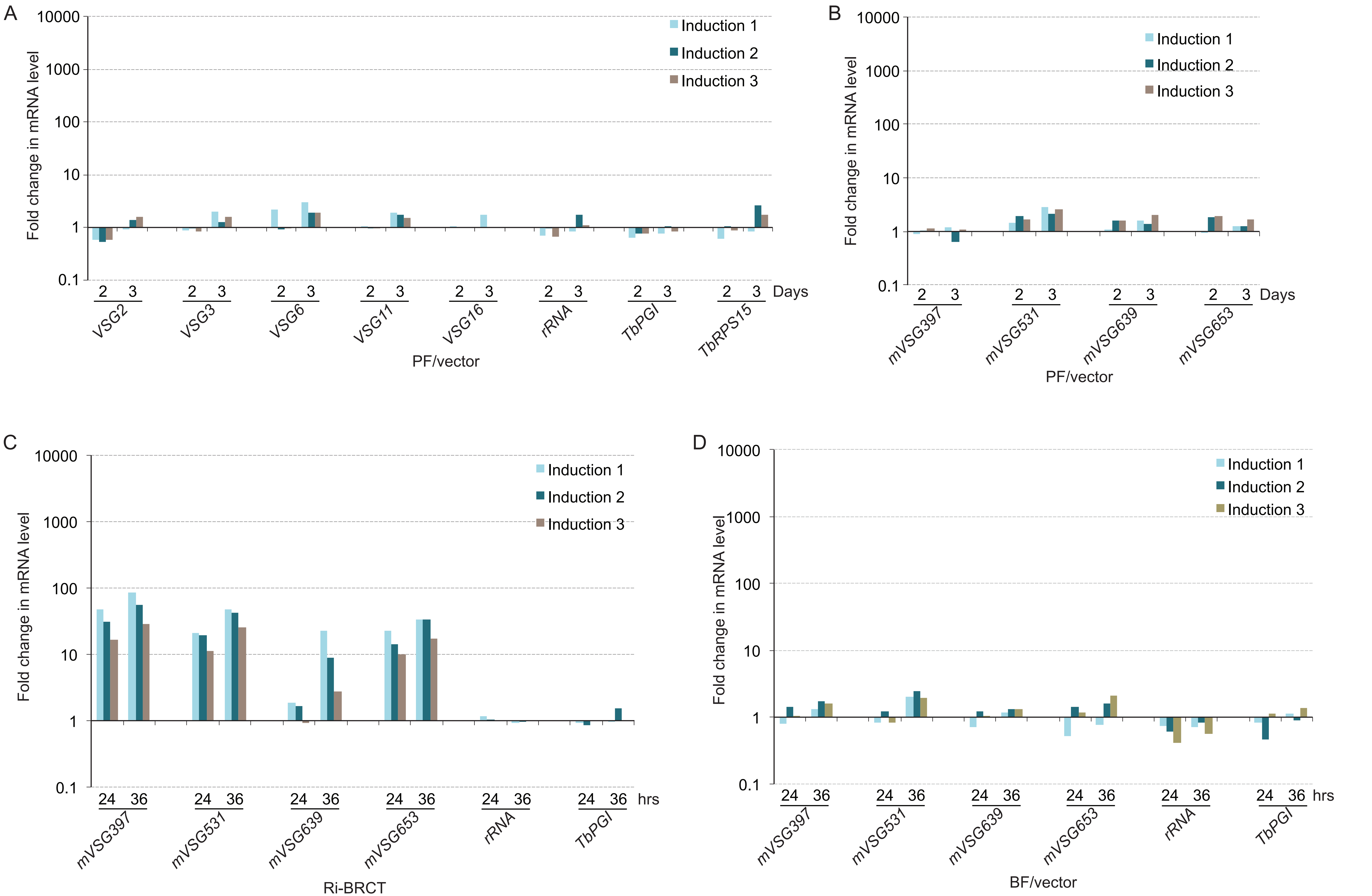
A
10000
1000
100
10
1
0.1
Fold change in mRNA level
Induction 1
Induction 2
Induction 3
2 3
Days
VSG2
VSG3
VSG6
VSG11
VSG16
rRNA
TbPGI
TbRPS15
PF/vector
B
mVSG397
mVSG531
mVSG639
mVSG653
PF/vector
C
24 36
hrs
mVSG397
mVSG531
mVSG639
mVSG653
rRNA
TbPGI
Ri-BRCT
D
BF/vector

Pandya et al.
Supplementary Figure 5

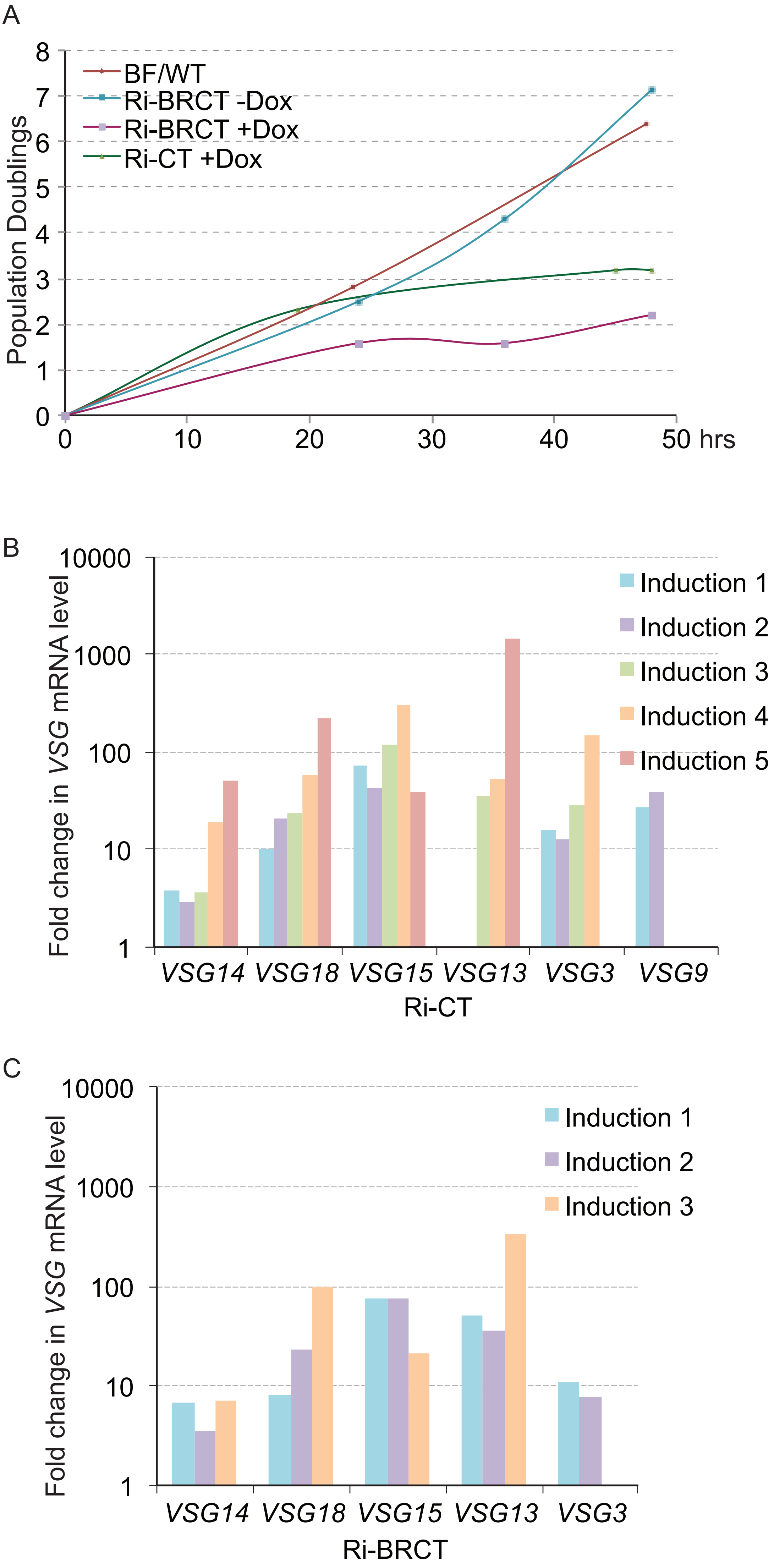

Pandya et al.
Supplementary Figure 6

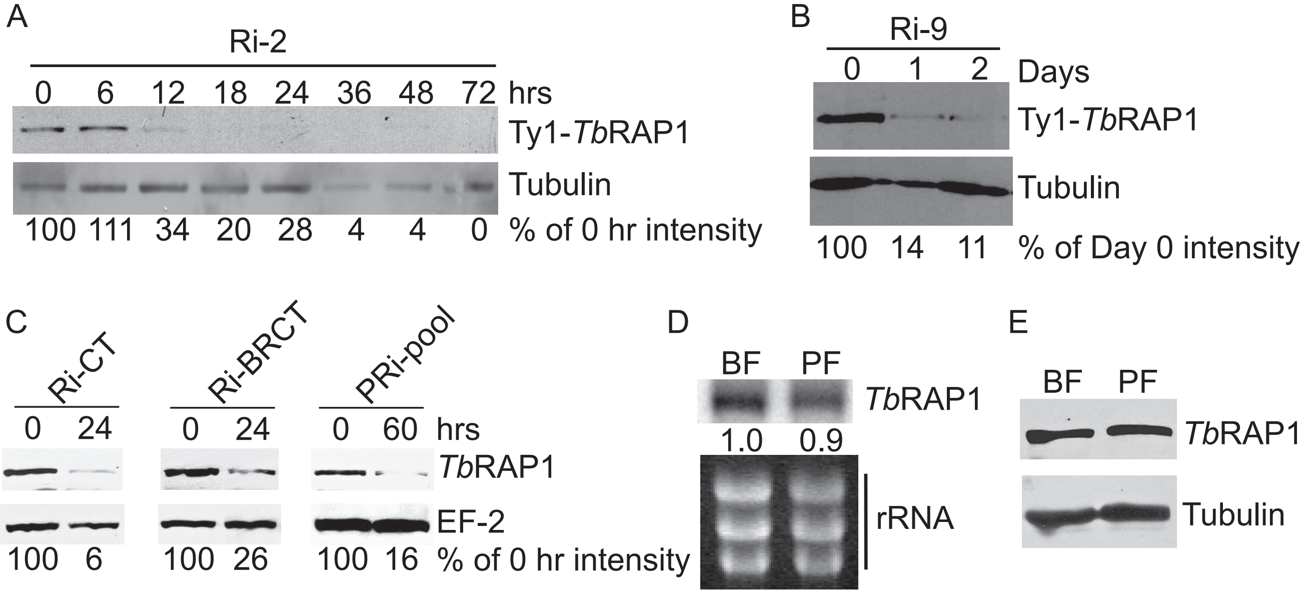

Pandya et al.
Supplementary Figure 7

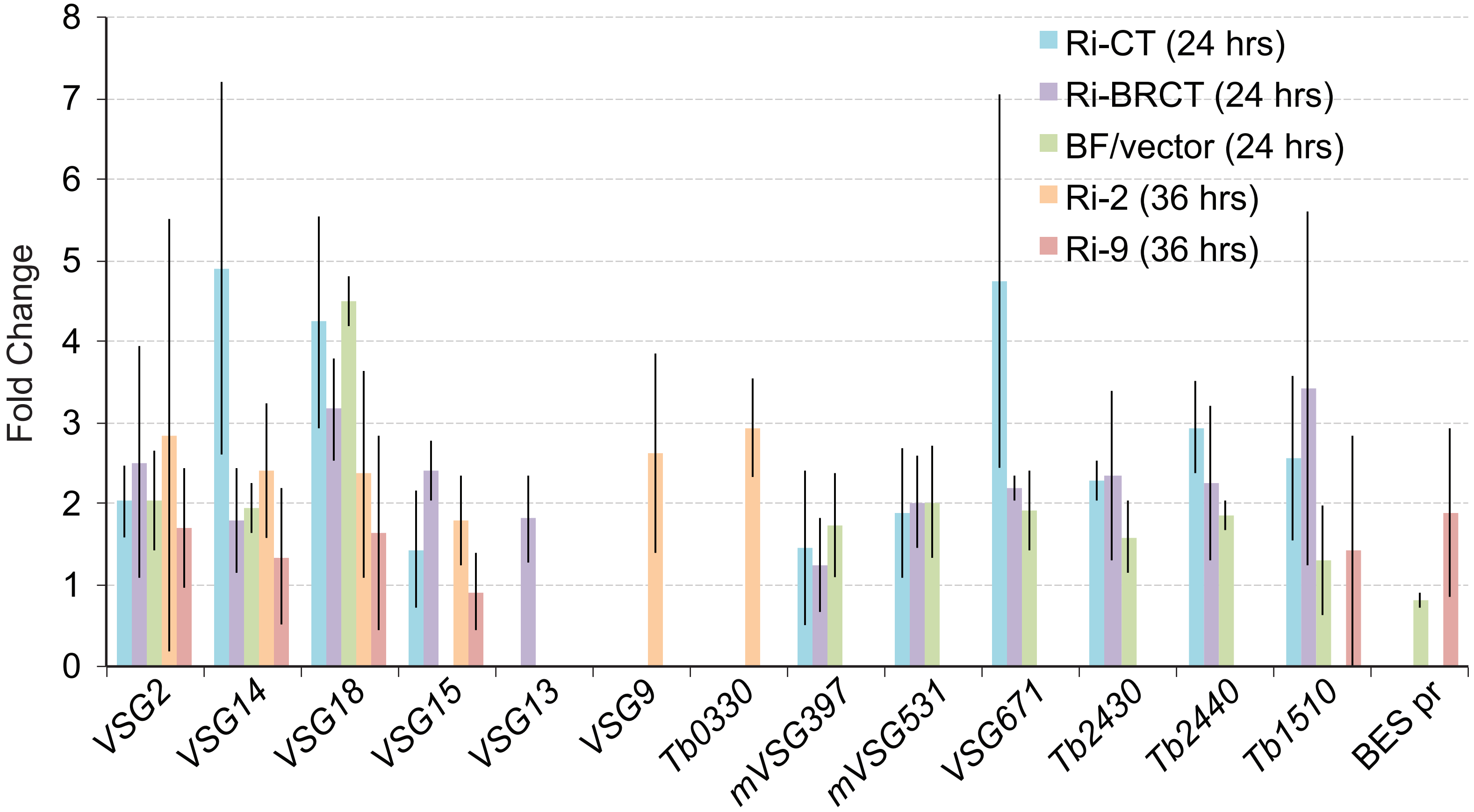

Pandya et al.
Supplementary Figure 8

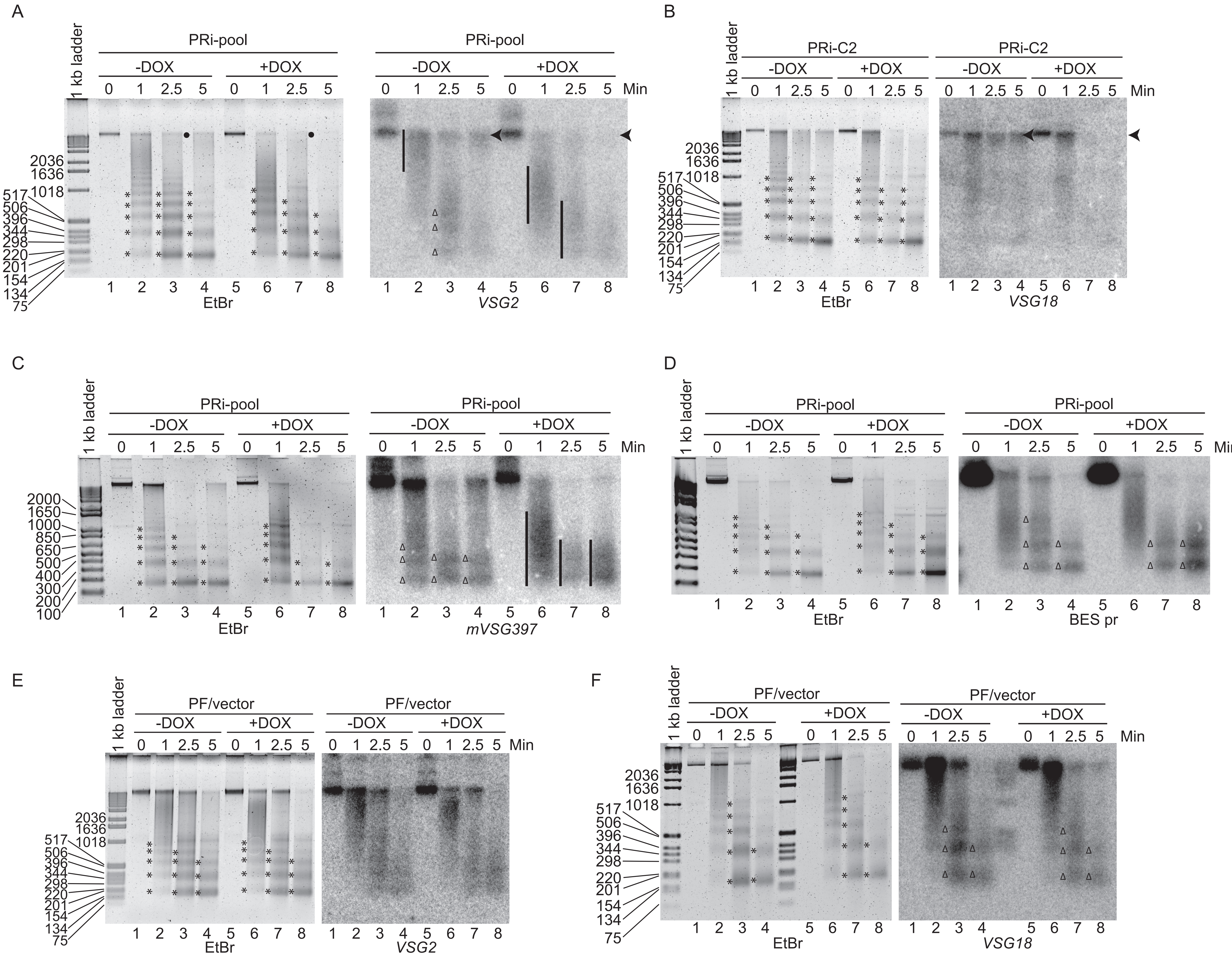

Pandya et al.
Supplementary Figure 9

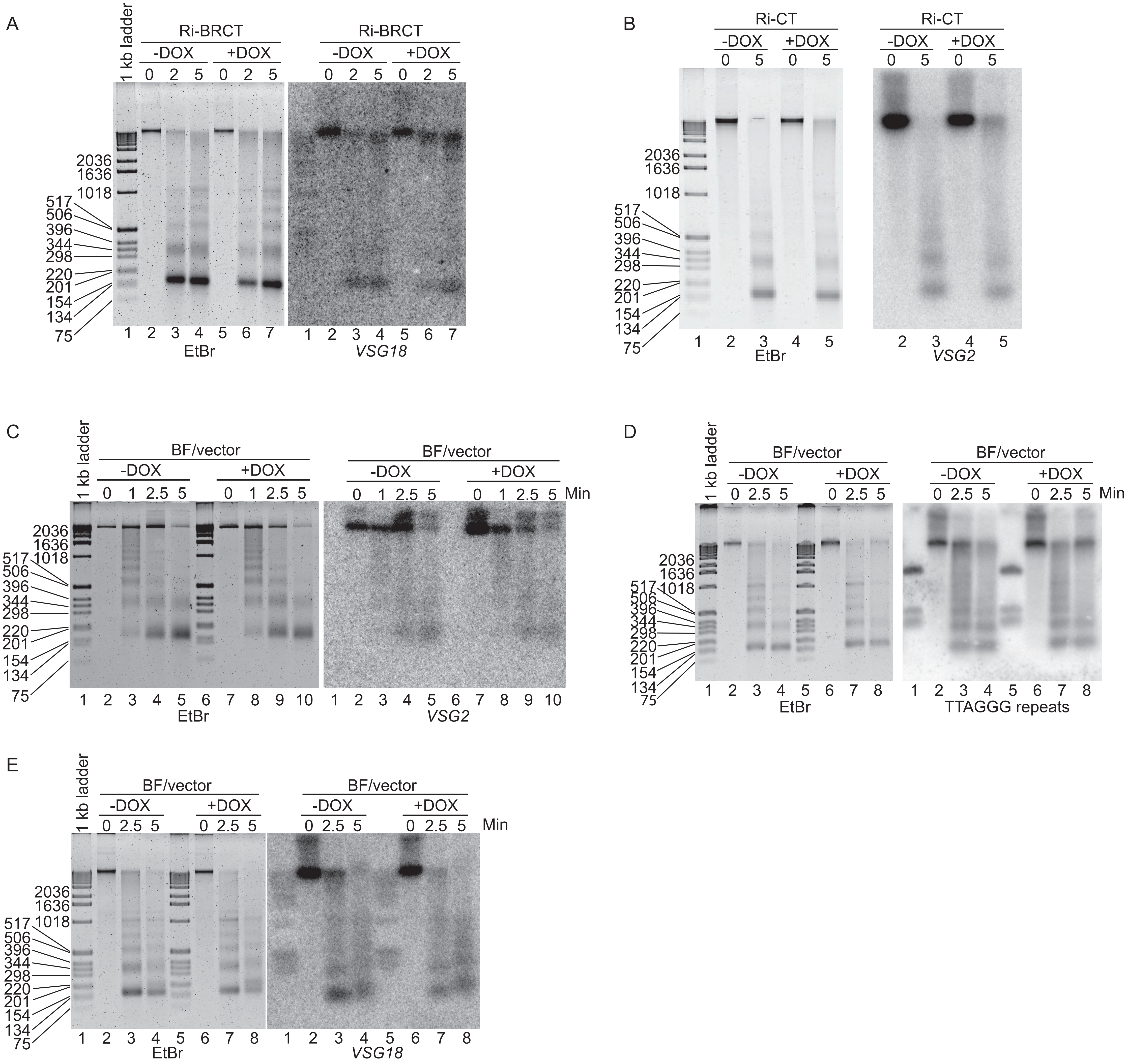

**Supplementary Table 1**

| VSG names used in this work | Aliases | *MITat* | BES |
|---|---|---|---|
| *VSG2* | *VSG221* | *MITat 1.2* | 1 |
| *VSG3* | *VSG224* | *MITat 1.3* | 7 |
| *VSG6* | *VSG VO2* | *MITat 1.6* | 3 |
| *VSG9* | *VSG121* | *MITat 1.9* | 2 |
| *VSG11* | *VSG bR2* | *MITat 1.11* | 15 |
| *VSG13* | *VSG NA1* | *MITat 1.13* | 17 |
| *VSG14* | - | *MITat 1.14* | 8 |
| *VSG15* | - | *MITat 1.15* | 10 |
| *VSG16* | - | *MITat 1.16* | 11 |
| *VSG18* | *VSG800* | *MITat 1.18* | 5 |

## Supplementary Table 2

| Cell line | Parent* or common names in published literature | Life cycle stage | integrated plasmid | Active VSG | Silent VSGs | References |
|---|---|---|---|---|---|---|
| BF/WT | SM | BF | none | VSG2 | VSG3, VSG9 | 56 |
| Ri-2 | SM* | BF | p2T7-TABlue-TbRAP1 | VSG2 | VSG3, VSG9 | 23 |
| pVS3-2/OD1-1 | SM* | BF | none | VSG9 | VSG3, VSG9 | 23 |
| Ri-9 | pVS3-2/OD1-1* | BF | p2T7-TABlue-TbRAP1 | VSG9 | VSG3, VSG9 | 23 |
| SM-derived line | SM* | BF | none | VSG3 | VSG2, VSG9 | This study |
| PF/WT | WT427 | PF | none | none | VSG2, VSG3, VSG9 | |
| PRi-pool | 29-13* | PF | pZJMb-TbRAP1-CT | none | VSG2, VSG3, VSG9 | This study |
| PRi-C2 | 29-13* | PF | pZJMb-TbRAP1-CT | none | VSG2, VSG3, VSG9 | This study |
| Ri-CT | SM* | BF | pZJMb-TbRAP1-CT | VSG2 | VSG3, VSG9 | This study |
| Ri-BRCT | SM* | BF | pZJMb-TbRAP1-BRCT | VSG2 | VSG3, VSG9 | This study |
